# Supplementary material for: Continuous Perfusion Experiments on 3D Cell Proliferation in Acoustic Levitation
Source: Micromachines (Basel). 2024 Mar 25;15(4):436. doi: 10.3390/mi15040436 (PMC11051894; doi:10.3390/mi15040436)
Supplement: Supplementary file 1 [file micromachines-15-00436-s001.zip › micromachines-2894314-supplementary.pdf]

<u>Supplementary Data</u>

Continuous Perfusion Experiments on 3D Cell Proliferation in Acoustic Levitation

Luca Fabiano [1,†], Shilpi Pandey [1,*,†], Martin Brischwein [1], Morteza Hasanzadeh Kafshgari [1,2,3] and Oliver Hayden [1,*]

[1] Heinz-Nixdorf-Chair of Biomedical Electronics, School of Computation, Information and Technology, Technical University of Munich, TranslaTUM, 80333 Munich, Germany; luca.fabiano@tum.de (L.F.); brischwein@tum.de (M.B.); morteza.kafshgari@tum.de (M.K.)
[2] Central Institute for Translational Cancer Research (TranslaTUM), School of Medicine, Technical University of Munich, 80333 Munich, Germany
[3] Department of Radiation Oncology, School of Medicine, Technical University of Munich, 80333 Munich, Germany
* Correspondence: shilpi.pandey@tum.de (S.P.); oliver.hayden@tum.de (O.H.)
† These authors contributed equally to this work.

1.1 The highlighted challenges of workflow solutions are as follows:

- The size regime of cell interactions can be studied with diffraction-limited microscopy without the need for labelling.
- Our platform allows comprehensive analysis along all axes within the culture space, enhancing the understanding of cellular behaviour in complex environments. Other platforms are often limited to cells attached to a surface, allowing analysis of 2D cells only.
- Low dead volume, high recovery rate, and a trapping time of less than one minute.
- Sufficient trapping force for continuous perfusion experiments and low shear stress induced by nutrient flow.
- A single robust and drift-free trap for actuator-free optical inspection, unlike random aggregates formed in, e.g. SAW-based traps, or other acoustic platforms, which have a stochastic pattern, and the position of trapped objects varies reducing repeatability.
- Moderately low cost for a cartridge as, unlike SAWs, no wafer processing is required, enabling transmission microscopy and minimal effort for closed-loop systems (shown for temperature control).

1.2 Cell Viability

As mentioned in the manuscript, some experiments were performed under an acoustic trap to check cell viability using PI staining (1 μL/mL). During the viability experiment, only a few cells died among other captured cells, which was carried out for 55 h, as shown in Figure S1. The image in Figure S1 (B and C) validates that only a few cells are dead, and most of the cells remain viable. There was a small percentage of dead cells in the cell suspension. We visually monitored the cells during the continuous acoustic trapping by taking a brightfield and fluorescence image every 5 min. Nutrients were supplied continuously for 55 hours at a pressure of 2 mbar, and the incubator temperature was also controlled at 37 °C.

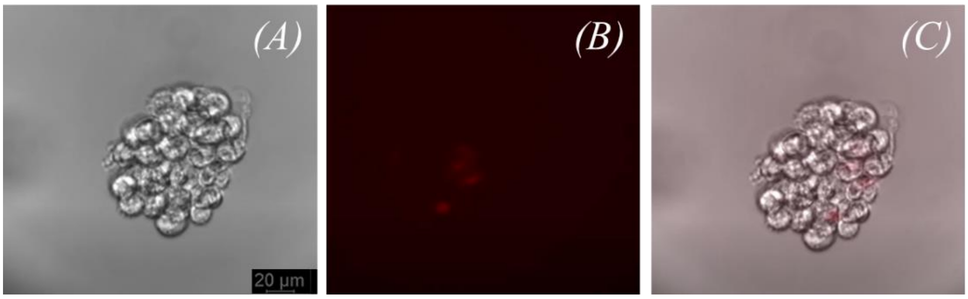

Figure S1. An experiment on the viability of the cells was carried out using an acoustic trap: (**A**) Bright field image of trapped cells at mode 1; (**B**) Fluorescent image of trapped cells, indicating that only a few cells are dead, whereas the majority is alive; (**C**) Overlay image, which shows dead (marked with red fluorescent-PI) and live cells in the trap.

The calculated live and death rates were ~95% and ~5% respectively. Cell viability, particularly in 3D cultures, was assessed primarily by the morphology and using a propidium iodide (PI) marker. The process involved an approximate calculation using live/dead PI staining.

The analysis of a 3D cell cluster, assuming a spherical shape with a diameter of approximately 65 µm and individual cells measuring around 15 µm, yielded an estimated total of ~81 cells within the cluster. It was determined that four cells were dead, leaving 77 cells alive. The calculated cell death rate was approximately ~5%, while the cell viability was approximately ~95%. The 3D cluster was assumed to be spherical and uniformly distributed.

1.3 Proliferation Experiment

Rotation was also observed during the degeneration of cells, as highlighted in the article's main text. Figure S2 exemplifies the degeneration of cells and rotation, which is induced because of cell death.

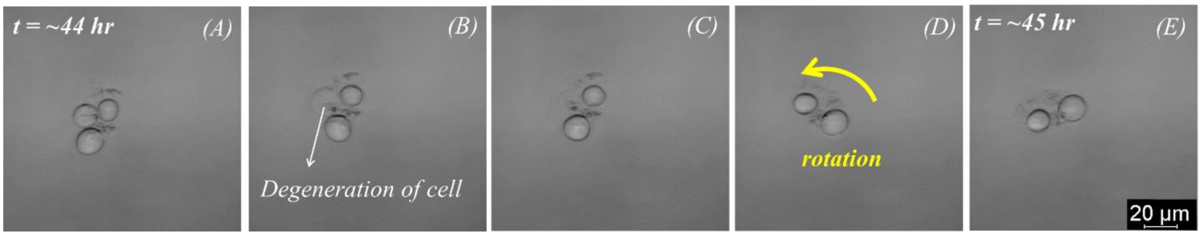

Figure S2: (**A**,**B**) Degeneration of cell and debris formation; (**C–E**): Rotation for about 60 min was observed right after the degeneration of cell.
